# Consideration of Critical Parameters for Improving the Efficiency of Concrete Structures Reinforced with FRP

**DOI:** 10.3390/ma15082774

**Published:** 2022-04-09

**Authors:** Krzysztof Adam Ostrowski, Carlos Chastre, Kazimierz Furtak, Seweryn Malazdrewicz

**Affiliations:** 1 Faculty of Civil Engineering, Cracow University of Technology, 24 Warszawska Str., 31-155 Cracow, Poland; kfurtak@pk.edu.pl; 2 CERIS and Department of Civil Engineering, Universidade Nova de Lisboa, 2829-516 Caparica, Portugal; chastre@fct.unl.pt; 3 Department of Materials Engineering and Construction Processes, Wroclaw University of Science and Technology, Wybrzeze Wyspiańskiego 27, 50-370 Wroclaw, Poland; seweryn.malazdrewicz@pwr.edu.pl

**Keywords:** FRP, concrete, CFRP, BFRP, GFRP, AFRP, cement matrix, epoxy resin, reinforcement, compressive strength

## Abstract

Fibre-reinforced polymer materials (FRP) are increasingly used to reinforce structural elements. Due to this, it is possible to increase the load-bearing capacity of polymer, wooden, concrete, and metal structures. In this article, the authors collected all the crucial aspects that influence the behaviour of concrete elements reinforced with FRP. The main types of FRP, their characterization, and their impact on the load-carrying capacity of a composite structure are discussed. The most significant aspects, such as type, number of FRP layers including fibre orientation, type of matrix, reinforcement of concrete columns, preparation of a concrete surface, fire-resistance aspects, recommended conditions for the lamination process, FRP laying methods, and design aspects were considered. Attention and special emphasis were focused on the description of the current research results related to various types of concrete reinforced with FRP composites. To understand which aspects should be taken into account when designing concrete reinforcement with composite materials, the main guidelines are presented in tabular form.

## 1. Introduction and Aim of the Technical Note

Nowadays, concrete is the most common building material [1]. More and more often, elements of the load-bearing structures with complicated shapes are made of advanced concrete, which was impossible until recently. However, there are concrete structures built several decades ago that still fulfil their role and often require repair and reinforcement. This is largely due to the much lower level of concrete technology a few decades ago, the low strength of concrete, and the course of the natural process of concrete carbonation. Additionally, in the 20th century, a previously unknown deleterious chemical reaction involving pore solutions in the concrete and certain compositions of siliceous aggregates was detected [2]. The resulting expansion, known as alkali–silica reactivity (ASR), could lead to abnormal cracking in a variety of patterns (depending on the design of the structure, reinforcement, detailing, restraints, and exposure conditions), reduction in strength, and early in-service failure of the concrete structure [3]. The alkali–silica reactions are responsible for the degradation of a number of structures and reinforcement corrosion. For instance, alkali–aggregate reaction is considered to be the second most important factor (after corrosion of reinforcing steel), causing premature destruction of concrete structures. As a result of the reaction of sodium and potassium hydroxides with reactive silica (which is part of some aggregates), a destructive process occurs that requires repair and potential reinforcement. In order to ensure further, safe exploitation of concrete structures, they can be reinforced with composite materials [4]. Nowadays, it is the most widely used technology that allows for effective reinforcement of reinforced concrete structures. An example of such reinforcement is sticking a tape, mat, or mesh to the surface of the reinforced element using an effective binder (usually epoxy resin). This technology does not disturb the architecture of the building due to the small influence of the glued reinforcement on the cross-section geometry of the concrete elements. A literature review from [5] confirmed the feature of FRC—crack control. This can limit the rate at which substances such as water, chlorides, and carbon dioxide ingress into structural elements, thereby prolonging the service life of the structure.

However, the correct process for strengthening concrete structures depends on a number of factors. The first factor is the choice of the type of FRP composite fibres taking into account the environmental impact to which the reinforced element is exposed. The second is the selection of the matrix co-creating the composite structure in which the fibres/mats/tapes will be embedded. Other equally important factors determining the quality of the reinforcement made are: the orientation of the fibres in the composite, the course of the lamination process, preparation of the concrete substrate, and protection of the composite against the effects of an aggressive environment such as physical and chemical factors. Bearing the above aspects in mind and the fact that the reinforcement of concrete structures with the use of composite materials has developed rapidly in recent years [6,7], the authors presented the most important factors influencing the effectiveness of structural strengthening. In the authors’ opinion, this technical note will be helpful to a wide range of concrete reinforcement contractors, providing necessary information on FRP from the design stage to installation. This can contribute to resolving the problem of concrete being required for reinforcement, thus ensuring satisfactory and safe performance of concrete structures.

## 2. Material Characteristics

The reinforcement of concrete structures with the use of composite materials has developed rapidly in recent years. In order to provide information on FRP for concrete reinforcement contractors, the authors provided a short description of composites and FRP composites. Examples of how FRP composites can be used in the civil engineering industry are presented.

### 2.1. Composites

**Figure 1:**
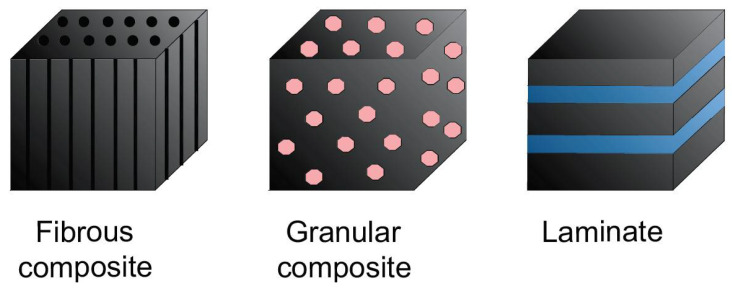
Classification of composites regarding their construction, adapted from [11].

Composites are based on two or more fundamental materials, and when combined, they have properties other than individual constituent materials. According to Rajczyk and Stachecki [8], composites can combine materials of the same type, as well as various types of materials, such as metals, polymers, and ceramics, using their specific characteristics in a thoughtful manner. In the case of particle-reinforced composites, one can distinguish two subtypes, depending on the particle size used. Dispersion particles are those which are connected to the matrix at the microscopic, atomic, or molecular level. In contrast to a composite reinforced with small particles, the most popular example of a composite reinforced with large particles (often called aggregate composite) is concrete. The continuous phase is then cement mortar, while the reinforcement is the aggregate. In such reinforced composites (compared with dispersively reinforced composites), the transfer of external loads is the result of a common matrix load capacity and a dispersed phase, in which independent rigidity and hardness are greater than the rigidity and hardness of the surrounding matrix. Therefore, the mechanism of interaction of particles with the matrix is also changed. Fibre-reinforced composites exhibit a favourable strength-to-weight ratio. Due to this, they are currently dominating the composite materials market due to their low weight and superior mechanical and strength properties [9,10]. The working principle of fibre composites is based on the transfer of loads through various types of fibres. The matrix serves only as a binder for the fibres and as a direct protection against external factors. The fibres used can be continuous or discontinuous (whiskers and cut fibres). Numerous products made of single fibres can also be used as reinforcement. Structural composites are complex materials with a homogeneous or mixed material structure, whose modernization and widespread use in industry have resulted in the development of the latest technologies. This group includes laminates, i.e., interconnected layers of two-dimensional composites and also layered composites, i.e., systems with a clear division of functions concerning strength and protection. The basic laminate layer (laminate) forms a resin-bonded fibre (single composite). The laminate itself is a system of interconnected composite layers (usually with different parameters) that are properly oriented with respect to the direction of the main load, to use the best possible arrangement of individual layers. The classification of composites regarding their construction is presented in Figure 1.

A composite consists of a matrix and a second component (reinforcement) placed in it, which has much better mechanical properties [12]. The major functions of the matrix are as follows:

- To maintain the entire system in a compact form (combines reinforcement);
- To transfer external loads to the reinforcement;
- To provide appropriate strength;
- To influence the chemical and thermal properties;
- To protect reinforcement against mechanical damage;
- To give products a specific shape.

The major functions of the inner reinforcement are:

- To improve strength properties;
- To increase resistance to abrasion;
- To reduce thermal expansion;
- To increase resistance to thermal shock;
- To stop the propagation of cracks;
- To increase the load-bearing capacity of structures.

### 2.2. FRP Composites

**Figure 2:**
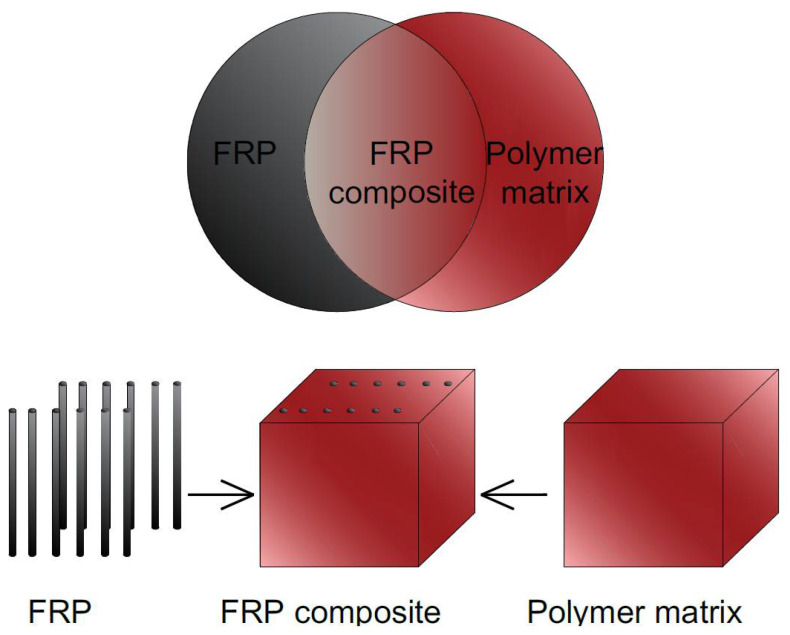
The idea of FRP composites, adapted from [17].

**Figure 3:**
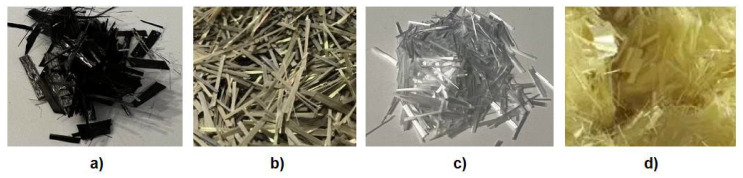
Main type of fibres: (**a**) carbon fibres; (**b**) basalt fibres; (**c**) glass fibres; and (**d**) aramid fibres.

Fibre-reinforced polymer (FRP) is a composite material made of a polymer matrix reinforced with fibres (Figure 2). Due to numerous benefits, the usage of FRP in concrete technology is becoming more and more popular to strengthen existing elements, but also to design new hybrid structures. The high Young modulus, strength to mass ratio, resistance to aggressive environments, good fatigue properties, low lifecycle costs, electromagnetic transparency, and low thermal conductivity are the major advantages of FRP [13,14,15,16]. Appropriate inclusion of steel or non-metallic fibres has been proven to increase both the tensile capacity and ductility of FRP concrete. The major fibres used as the reinforcement in composites are carbon, basalt, aramid, and glass fibres (Figure 3). The most commonly used laminates in civil engineering are: carbon fibre polymer materials (CFRP), basalt fibre polymer materials (BFRP), aramid fibre polymer materials (AFRP), and glass fibre polymer materials (GFRP).

### 2.3. The Importance of FRP Materials in Civil Engineering

Steel, concrete, and wood are the basic structural materials that are widely used in civil engineering. The FRP technique could be perfectly adapted to improve the properties of these materials and change the load-bearing capacity of structural elements. FRP materials can be used for various types of reinforcements. There are no significant restrictions in the strengthening of elements with complex geometry such as beams, chimneys, columns, walls, columns, silos, and supporting structures for pipelines and gas pipelines. Due to their properties (good tensile strength, ductility, and fatigue resistance), FRPs have been used in a wide range of applications, including pavements, industrial floors, tunnel linings, slope stabilization, impact-resistant structures, and structures sensitive to earthquakes [5]. Increasing the durability leads to longer service life, thus achieving a reduction in the overall environmental impact of the element over its entire lifecycle. The very low weight of polymer fibre mats is extremely beneficial when reinforcing structural elements. The analysed method of strengthening the structure, in comparison with traditional methods, is beneficial in the long term.

FRP materials can be used in reinforced steel, concrete, and wooden and brick constructions to:

- Increase resistance to the seismic loads of masonry structures;
- Increase the strength of concrete and reinforced columns;
- Replace missing reinforcements;
- Enable the reassignment of buildings;
- Increase the strength of individual elements and the entire structure;
- Improve the load capacity of a structure that is weakened due to structural errors;
- Adapt the load capacity of a structure to the applicable standards and requirements;
- Enhancement of service loads;
- Increase service life and durability;
- Improve resistance to seismic loads.

## 3. Factors Influencing Strengthening of Concrete Structures with FRP Composites

The correct process for strengthening concrete structures depends on a number of factors. From the choice of the type of FRP composite fibres and the matrix up to lamination process, the authors presented the most important factors influencing the effectiveness of structural strengthening.

### 3.1. Type of Fibres

The mechanical and physical properties of the most common fibres used in FRP laminates are presented in Table 1.

Carbon fibres (CF) are one of the most common fibres used to reinforce engineering structures, and are characterized by chemical and thermal resistance. One of the major important aspects regarding CF is the permissible temperature to use them up to even 2000 °C, in contrast to glass or aramid fibres [31,32]. Acceptable temperatures for the possible use of different types of fibres are given in Table 2.

Basalt fibres (BF) are produced by melting crumpled basalt rocks at a high temperature equal to 1400 °C. BF have better physical and mechanical properties than glass fibres. The cost of BF is lower than CF and much more expensive than glass fibres.

Aramid fibres (AF) are characterised by excellent chemical, mechanical, and physical properties at high temperatures. Unfortunately, AF have a very low resistance to ultraviolet light. Under the influence of long sunlight, the strength properties of these fibres may drop by more than 50%. Kevlar fibres and modified AF with chains including p-disubstituted benzene are characterised by much better mechanical properties than standard AF.

**Figure 4:**
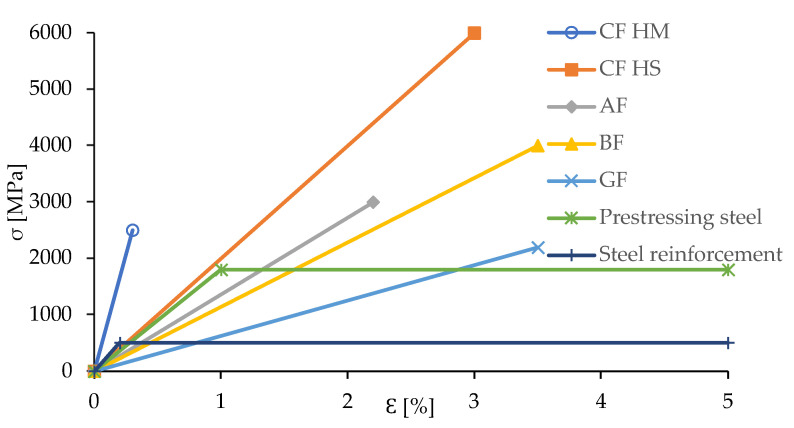
Stress–strain characteristics for fibre materials and steel, adapted from [33].

Glass fibres (GF) are chemical fibres obtained from water glass, and sometimes also from melted glass. Changes in the amount of raw materials, such as clay for alumina, sand for silica, colemanite for boron oxide, or calcite for calcium oxide enable the obtainment of different types of glass fibres. The advantages of GF are mainly their high mechanical strength, lightness, and resistance to aggressive environments. GF are available in a variety of forms and shapes, which is why they are widely used in engineering. The stress–strain characteristics for FRP materials and steel are presented in Figure 4. As shown in Figure 4, all these fibres have a higher tensile strength than common reinforcing steel and prestressing steel. However, it should be emphasized that one of the basic material parameters determining the use of a given type of fibre is its stiffness. It is only in the case of carbon fibres that their modulus of elasticity is higher than that of both steels. Combined with the highest tensile strength of carbon fibres, they are the most widely used in civil engineering. It is also worth noting that the deformability of fibres is lower than of steel. The CF HM fibres stand out here, for which deformability is very low and stiffness is significant.

### 3.2. Orientation of Fibres in FRP Composites

**Figure 5:**
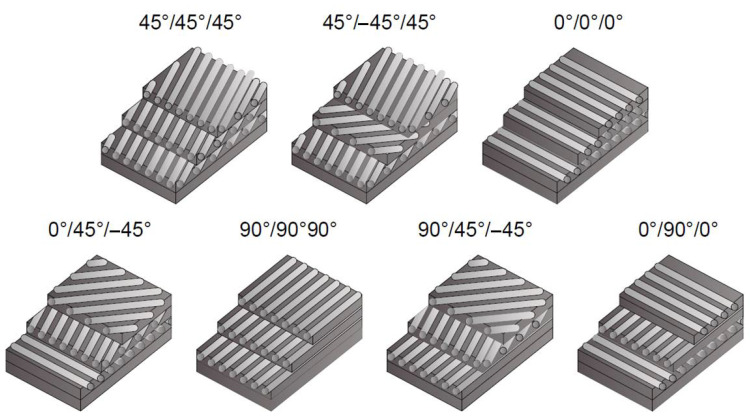
Different orientations of carbon fibres in composite manufactured from three CFRP laminates, adapted from [36].

The orientation of fibres in FRP composites has the most significant influence on the load-bearing capacity of reinforced concrete structures. Creating mats from carbon fibres allows the fibres in the mats to be freely arranged and different weaves to be created. For ordered fibres, the material properties are strongly anisotropic and the Young’s modulus and strength are a function of the volume fractions of the fibres and the matrix. In addition, the modulus of elasticity and strength in the ordering direction are much greater than in the direction perpendicular to the ordering. If the fibres are arranged in an orthogonal way, there are directions of ordering and disorder called anisotropy. In this case, the best mechanical properties occur under load along the fibres. Disordered fibres are characterized by isotropic properties and an ultimate breaking strength lower than in the case of materials with ordered fibres. The different orientation of carbon fibres in composite manufactured from three CFRP laminates is presented in Figure 5. Depending on the stress maps occurring in reinforced concrete structures, FRP mats with an appropriate weave should be selected, which will ensure optimal behaviour of the structure. Bhatnagar et. al. [34] presented the study about understanding the effects of the fibre orientation and the machining direction on the cutting behaviour with unidirectional carbon-fibre-reinforced plastic (UDCFRP) laminates. The various fibre orientation angles chosen for observing the chip formation behaviour and cutting forces were 0°, 10°, 30°, 45°, 60°, 75°, and 90° at two different rake angles, 12° and 18°. The results from Table 3 show that minimum force occurs in the range of 0° to +30° fibre orientation. The values of cutting force on the negative side are larger than their counterparts. The resultant cutting forces are much higher in the negative cutting direction; the maximum value reaches the range of −30° to −60° fibre orientation. There is approximately a 100% growth in the resultant forces when the direction of machining is reversed from +0 to −0. Frangopol and Recek [35] concluded that each layer of laminate has a different strength due to its fibres’ orientation and, therefore, a different reliability. The presence of an additional layer in a composite laminate plate does not necessarily increase the reliability and can increase the probability of failure of other layers. It is necessary to consider layer-interaction effects in evaluating the reliability of FRCs and fibre orientation.

### 3.3. Lamination Process

Every contractor knows that proper installation is the key process, no matter the technology. This paragraph will be especially useful for contractors, seeking information on FRP installation. The efficiency of strengthening structural elements depends on the correct fibre lamination process. There are two methods for carrying out the process of strengthening engineering objects using FRP fibres and epoxy resin: the wet lay-up and the dry lay-up.

**Figure 6:**
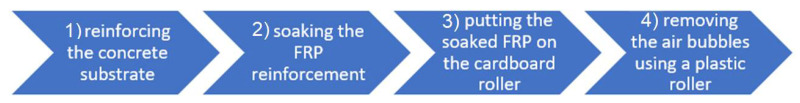
Stages in the wet lay-up process, adapted from [37].

The name ‘wet lay-up method’ comes from the status of the FRP at the time of application in its final position. In this method, part of the epoxy resin is applied directly to the FRP reinforcement, while the other part of the resin, with a thixotropic agent, is used to reinforce the concrete substrate. At the beginning, the structural element should be reinforced with epoxy resin with a thixotropic agent (Figure 6(1)). The resin can be applied mechanically using a saturator, or manually using a roller, trowel, or brush. The next step is to soak the FRP reinforcement with epoxy resin using a roller (Figure 6(2)). Rolling should take place along the FRP fibres. The effectiveness of the polymer’s cooperation with the reinforcement depends on the correct soaking of the FRP mats. The easiest way to transfer a wet FRP mat is to put it on a cardboard roller (Figure 6(3)). Thanks to this, all deformations of the FRP can be eliminated. The FRP should then be glued to the previously prepared concrete substrate. It is important to properly arrange the FRP so that a proper direction of fibres is established. In the final stage, the laminate surface is smoothed and air bubbles from the FRP composite are removed (Figure 6(4)) using a plastic roller (rolling along the FRP fibres). 

**Figure 7:**
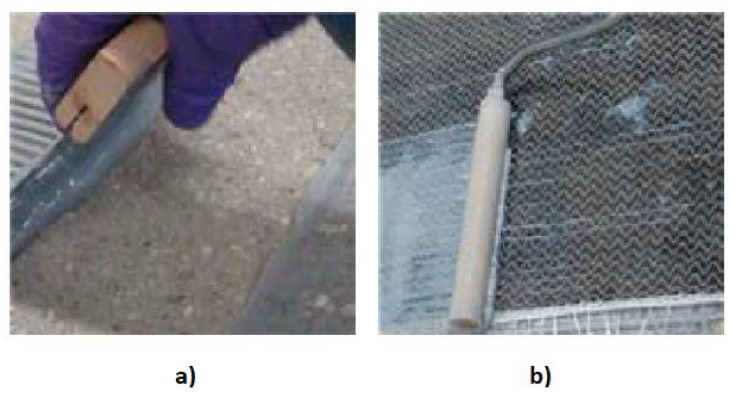
Stages in the dry lay-up process: (**a**) reinforcing the concrete substrate and (**b**) applying the FRP reinforcement to the concrete substrate, adapted from [27].

The name ‘dry lay-up method’ also comes from the status of the FRP at the time of application in its final position. In this method, the epoxy resin is used to reinforce the concrete substrate and to impregnate the FRP. Firstly, epoxy resin is applied to the concrete element using a trowel, brush, or roller (Figure 7a). The next step is to apply the previously cut FRP reinforcement to the concrete element and laminate the FRP using a plastic roller along the fibres (Figure 7b). If the application of more than one layer of FRP is planned, this should be done using the ‘wet on wet’ method (applying the next layer of FRP on the previous one, before the material dries). If not, a period of at least twelve hours (due to the hardening process of epoxy resin) must pass before the next surface of the composite is applied.

### 3.4. Matrix

**Figure 8:**
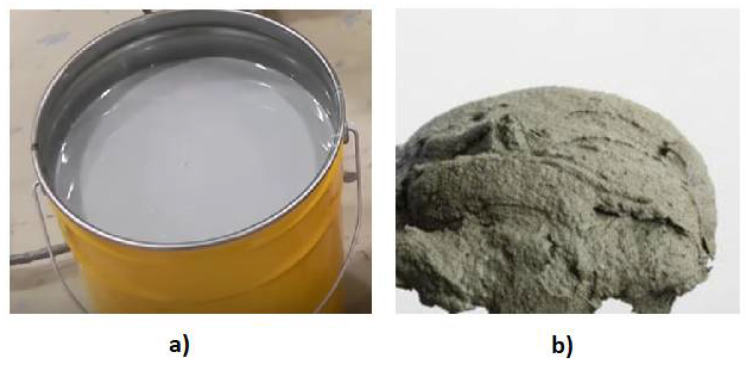
Example of the matrix in the laminate: (**a**) epoxy resin and (**b**) cement.

The adhesion between the matrix and reinforcement has a crucial role during the transfer of loads [38]. The matrix in the laminate is formed by epoxy resin (Figure 8a) or, less frequently, by the cement matrix (Figure 8b). Epoxy resin consists of two components: resin and hardener. After combining the ingredients in the right proportions, the working time for applying the resin is about one hour at +20 °C, which decreases with an increasing temperature (due to the viscosity of the resin increasing rapidly and its gelation). The hardening of the resin is accompanied by the formation of a large amount of heat. Epoxy resin is a liquid polymer material, which has been used in industry for over 50 years. It is used as a matrix to reinforce structural elements made of concrete, metals, and wood. Due to its properties, it works effectively with the mentioned materials. Epoxy resin can be applied in all cases of reinforcing structural elements with FRP materials.

One of the major drawbacks of epoxy resin-based composites is plastic behaviour at low temperatures. Due to this fact, a modified cement matrix has been considered as a potential alternative in recent years. This specific cement-based matrix is less sensitive to temperature changes in comparison with epoxy resin. Furthermore, the matrix could be internally reinforced with natural fibres which could improve the properties of the raw matrix [39]. The cement matrix consists of high-quality cement (especially Portland cement types I–CEM I 42,5R or CEM I 52,5R), silica fume, superplasticizer, and water. The efficient time for usage of both the epoxy resin and the cement matrix (from the moment the ingredients are mixed) is similar and mainly depends on ambient temperature. Unfortunately, adhesion of the cement matrix to some types of concrete is practically zero, so its use is strongly limited to low-strength concrete. The advantages and disadvantages of epoxy resin and cement matrix are presented in Table 4.

### 3.5. Preparation of Concrete Substrate

**Figure 9:**
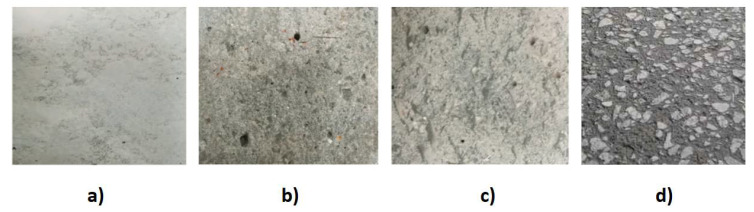
Types of concrete substrate surface: (**a**) unprepared, (**b**) sanded, (**c**) grinded, and (**d**) shot blasted.

Before the lamination process, concrete substrate (CS) should be prepared in a proper way. CS must be dry, clean, and free of surface moisture. The laitance, dust, curing compounds, oils, waxes, foreign particles, impregnations, and other coatings must be removed from the concrete surface. For the best results, sandblasting, grinding or shot blasting are recommended. An unprepared concrete surface is presented in Figure 9a.

Grinding is surface finishing with abrasive tools, which results in high dimensional and shape accuracy, as well as low roughness. The material from which grinding wheels are made of is most often diamond, corundum, boron carbide, or silicon carbide. Sandblasting is a technological process that consists of cleaning or shaping the surface with abrasive material (sand) in a stream of compressed air or liquid. The sanding effect is similar to grinding; however, the surface being cleaned is more even and obtains the required roughness. Shot blasting is a similar technology to sanding. In this method, instead of sand, special metal shots are used (in the form of metal spheres). Most often, the shots can be recovered and reused. Research was carried out by the authors [40] to investigate unprepared, grinded, and sanded concrete substrate (Figure 9b–d) and its result on the effectiveness of reinforcing concrete elements with FRP materials. It was found that mechanical surface treatment improves the adhesion of CFRP to the concrete substrate. The highest compressive strength and deformability of the concrete elements was demonstrated for the reinforced CFRP samples with a grinded concrete surface and the lowest for the unprepared one. However, the differences in the tested high-performance concrete are not large and concern only several percentages.

### 3.6. Concrete Surface Moisture and Temperature during Lamination

The humidity of concrete surfaces in the lamination process is one of the key factors affecting the quality of the concrete substrate and it further affects the joint between the main structure and the outer reinforcement. Before commencing the process of strengthening the structure, it is necessary to define the moisture content of the concrete substrate, and determine the dew point temperature (DPT) and relative humidity. Most manufacturers of composite materials and resins specify the maximum substrate moisture content to be 4–5% by weight. The temperature, however, must be at least a few °C above the DPT. Too high humidity of the concrete surface may reduce the penetration depth of the epoxy resin and reduce the adhesion of the binder to the concrete.

It is also very important that the resin should be at the ambient temperature where the lamination takes place and during the process. Adverse changes may occur in the laminate, which may result in its permanent under-hardening at temperatures below 10 °C. At temperatures above 40 °C, the resin gels too quickly, preventing the FRP fibres from oversaturating.

### 3.7. High-Temperature Protection

Due to the major disadvantage of epoxy resin, i.e., low resistance at high temperature, structural elements reinforced with an organic matrix should be protected against direct impact of high temperature and fire. Damage due to fire/high temperatures is one of the major destructive aspects that cause deterioration of reinforced concrete structures [41] and concrete reinforced with FRP technique.

The main reason for protecting the reinforced concrete structure against the effects of sunlight and elevated temperatures is the relatively low glass transition temperature of most epoxy resins, which ranges from 40 °C to 50 °C [42]. This leads to a change in the state of the epoxy resin, which goes from solid to plastic. Even slight deformation of the adhesive can lead to local loss of adhesion of FRP fibres to the epoxy matrix, leading to local stress relaxation. As a result, the composite may be destroyed and the bearing capacity of the structural element may be lost. It should be remembered that even black coloured carbon fibres (heat accumulating) covered with a transparent epoxy resin can contribute to the destruction of the composite. Due to the influence of sunlight, the layer can be significantly heated, which will stop fulfilling the role of the composite structure. As it has been shown in [43], even in the temperate geographical zone, the temperature of the adhesive under the thin laminate can reach 65 °C, which is 20 °C higher than the glass transition temperature of the most common epoxy-based adhesives on the market.

The main way of protecting structural elements is by properly insulating them using non-combustible mineral wool or cement mortar. A five-centimetre layer of mineral wool on an FRP composite with epoxy resin can protect the FRP composite from a rising resin temperature above the glass transition temperature for 120 min. A layer of cement mortar of a few centimetres on the FRP composite provides fire protection for a minimum of 240 min [44].

### 3.8. Non-Destructive Testing of FRP Laminates

**Figure 10:**
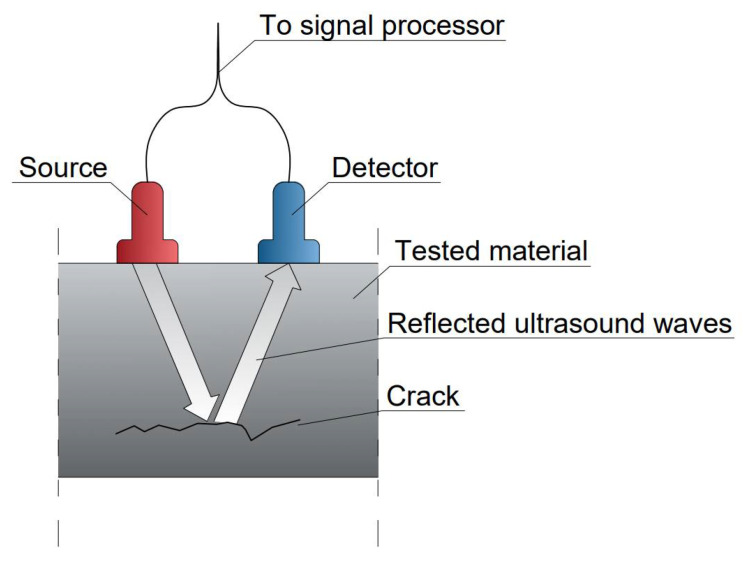
The idea of ultrasonic testing of material, adapted from [47].

Ultrasonic testing involves introducing ultrasonic waves into the object, which are reflected by material discontinuities, as well as bent and scattered on their edges (Figure 10). The purpose of the test, depending on the type of waves used, is to detect internal, surface, and subsurface discontinuities, to detect a lack of adhesion in glued, welded, soldered, and riveted joints, and to determine the properties of the materials [45,46]. The test allows the detection of flat and spatial discontinuities, internal and surface cracks, as well as inclusions and delamination. Due to the complex construction of composite structures, only this method is effective for assessing the state of FRP composites.

## 4. Concrete Reinforced with FRP Composites

In scientific publications, a lot of information can be found on the effectiveness of reinforcing concrete elements using FRP composites in the form of sheet, with regards to the strength of concrete. However, the strengthening may vary depending on the concrete used. The authors reviewed the literature concerning FRP composites on concrete with the division into concrete and FRP type. Table 5 shows selected results that, according to the authors, are selected due to their scientific importance and future perspectives. Laminate types were divided into four major categories: CFRP, BFRP, GFRP, and AFRP. The types of concrete involved: normal concrete, high performance concrete, ultra-high performance concrete, lightweight concrete, fibre-reinforced concrete, and recycled tire rubber concrete. All experiments were performed using small concrete column elements.

It has been proven that, as the number of reinforcement layers increases, the strength of the elements increases. Furthermore, as the compressive strength of concrete decreases, the effectiveness of the reinforcement increases.

Analysed research confirmed that epoxy resin is more commonly used than cement matrix, due to load-bearing efficiency (adhesion properties in the case of the cement matrix is low).

As the number of FRP laminates increases, the load-bearing capacity of a reinforced concrete structure increases. However, the research showed that three layers are enough to obtain 100% or more greater compressive strength in comparison with the reference concrete.

Technological parameters and their major influence on the efficiency of reinforcing concrete structures with FRP laminates are presented in Table 6. The authors collected the most significant aspects that have impact on the reinforcement. As seen below, to obtain the desired properties starts with the concrete used in the construction or design of the new one. The design stage of laminates includes determining their type, number of layers, direction of fibres, and the type of matrix. Proper installation of the reinforcement, including lamination in specified conditions and preparation of the concrete substrate are extremely important for the performance of the composite.

## 5. Durability of Concrete Structures Reinforced with FRP Materials

Fibre-reinforced polymer materials (FRP) are increasingly used to reinforce structural elements [66]. Due to this, it is possible to increase the load-bearing capacity of polymer, wooden, concrete, and metal structures [67,68]. However, because FRP laminates are still a relatively new solution (depending on the region), there is not enough information on their long-term behaviour which is crucial due to the design process [69]. Still, the high demand of FRP structures in civil engineering raises the interest in their behaviour over time, or in other words, their durability. Durability can be defined as the intensive correlation between the maintenance of a material and its features, and the ability to resist technical wear and chemical actions. The aging of materials due to exploitation and environment impact results in the process of gradual durability loss [70]. If there is still insufficient information about long-term properties of FRP reinforcements, the design codes cannot consider durability factors properly.

In terms of technical wear and resisting chemical actions, carbon fibre-reinforced polymer composites seem to be the most suitable. They are insensitive to chloride ions (the same as aramid fibre-reinforced polymer composites) and have better fatigue strength due to no water absorption (the same as glass fibre-reinforced polymer materials, which is the cheapest solution). They are less durable than others, due to high chemical sensitivity to an alkali environment. Glass and aramid can hydrolyse, especially in the presence of the high alkalinity in concrete. The interface between the reinforcement and concrete also plays a great role in terms of durability. Resin-dominated mechanisms are responsible for the transfer of shear and transverse forces, thus effecting the bond [71]. However, fibres in general do not rust like steel does and are resistant to attack by chlorides [72]. The research simulating the corrosion in large-scale reinforced columns and their repair using CFRP [73] confirms this theory. Columns wrapped using CFRP were characterised by improved strength and a slow rate of post-repair corrosion. Extensive, post-repair corrosion resulted in no loss of strength or stiffness and only a slight reduction in the ductility of the repaired member.

According to the American Concrete Institute (ACI 440.2R-08) [74], the ultimate tensile strength of FRP laminates *f_fu_* and the effective strain Ɛ*_fd_* to prevent immediate debonding failure can be determined as follows:

(1) $$f_{fu}=C_{E}f_{fu*}$$

(2) $${Ɛ}_{fd}=0.41\sqrt{\frac{{f^{\prime }}_{c}}{nE_{f}t_{f}}\leq 0.9{Ɛ}_{fu}}$$

where:

- $C_{E}$—environmental factor;
- $f_{fu*}$—ultimate tensile strength of FRP material reported by manufacturer;
- ${f^{\prime }}_{c}$—specified compressive strength of concrete;
- n—number of layers of FRP reinforcements;
- $E_{f}$—tensile modulus of elasticity of FRP;
- $t_{f}$—nominal thickness of one layer of FRP reinforcement.

As shown in Table 7, durability reduction factor and long-term stress limitation factor introduced for bond capacity, in case of CFRP are 0.95 and 0.55, respectively. This leads to the conclusion that the preparation of the contact surface and the adhesion of the FRP reinforcement affect the durability.

## 6. Conclusions

Concrete structures built several decades ago often require repair and reinforcement. In order to ensure their further, safe exploitation, they can be reinforced with composite materials, which have developed rapidly in recent years. This paper discussed the main factors affecting the efficiency of reinforcing concrete structures with FRP materials. The authors presented the most important factors influencing the effectiveness of structural strengthening.

Section 2 contains basic characteristics of composites and FRP laminates of different technologies, and usage examples of FRP materials in civil engineering. In Section 3, some parameters affecting the strengthening are presented: the choice of the type of FRP composite fibres, the selection of the matrix co-creating the composite structure, orientation of the fibres, lamination process, preparation of the concrete substrate, and protection of the composite against the effects of high temperature and non-destructive testing of FRP laminates. Section 4 focuses on the influence of FRP composites on the strengthening level of concrete, depending on the concrete and FRP types used. Here, compressive strength of the reference concrete and the reinforced one of different types was compared. Technological, material and environmental parameters having impact on reinforcing concrete structures with FRP laminates were presented. In Section 5, some examples of durability of FRC structures for concrete are shown.

Limitations in the current paper result from the analysed reinforced material, which is concrete. Therefore, the work focuses on the most important aspects that must be taken into account by designers of concrete structure reinforcements. The authors summarized the applications of various types of FRP materials in concrete structures. This technical note will be helpful to a wide range of concrete reinforcement contractors, providing necessary information on FRP from the design stage to installation. This can contribute to ensuring further, satisfying and safe performance of concrete structures. Bearing in mind a responsible approach to sustainable construction, in a future work, the authors wish to focus on the analysis of the use of recycled polymer fibres for the reinforcement of concrete structures. This research will undoubtedly follow the current global trend in which the re-incorporation of waste materials into the structure is desirable.

## Figures and Tables

**Table 1:** Properties of the major fibres used in FRP composites, adapted from [18,19,20,21,22,23,24,25,26,27,28,29,30].

Fibre	Type	Young Modulus (GPa)	Tension Strength (MPa)	Ultimate Elongation at Break (%)	Density (kg/m^3^)
Carbon	High strength	200–280	2500–5500	1.5–2.2	1800
	High modulus	330–490	2100–2800	0.7–1.0	2000
Basalt	-	90–110	4000–4840	2.25–3.1	2600–2800
Glass	S-glass	86–93	4500–4890	1.93	2460–2490
	E-glass	72.3	3345–3400	2.12	2540–2580
Aramid	Kevlar 29	70–83	2900–2920	3.50–3.60	1440
	Kevlar 49	124–151.7	2758–3010	2.4	1467
	Kevlar 149	179	3450	1.3–1.6	1470

**Table 2:** Permissible temperatures when using fibres, adapted from [13].

Temperature Range	Type of Fibre
Low temperature (below 100 °C)	All available fibres: natural, glass, carbon, ceramic, boron, organic, metal
Raised temperature (100 °C–400 °C)	Some organic, glass, carbon, ceramic, boron, metal
High temperature (400 °C–700 °C)	Ceramic, metal, carbon
Very high temperature (above 700 °C)	Carbon, ceramic

**Table 3:** Cutting force based on fibre angle, adapted from [34].

Fiber Angle (°)	Tool Rake Angle			
	12°		18°	
	Cutting Force F_c_ (N)	Resultant Force F_t_ (N)	Cutting Force F_c_ (N)	Resultant Force F_t_ (N)
−75	162	110	170	65
−60	200	155	175	115
−45	160	181	160	170
−30	145	200	145	207
−10	135	210	140	215
0	145	140	120	125
10	120	120	105	85
30	115	40	155	45
45	140	62	105	85
60	158	61	215	45
75	140	60	170	40
90	315	110	300	98

Note: Cutting speed = 1.18 m/min; width of cut = 2.2 mm and depth of cut = 0.25 mm.

**Table 4:** Advantages and disadvantages of epoxy resin and cement matrix.

Matrix	Advantages	Disadvantages
Epoxy resin	Very good strength properties Compressive strength of 40–90 MPa Tensile strength of 12–40 MPa Bending strength of 20–60 MPa Minimal shrinkage during curing High chemical resistance to most solutions of inorganic and organic acids, hydroxides, and solutions of inorganic salts High hardness, abrasion, and scratch and impact resistance	No resistance to UV radiation Low elasticity Low chemical resistance to oxidizing substances, alcohols, hydrocarbons, and ketones Moisture sensitivity during implementation No resistance to high temperatures (plasticization at temperatures from 70 °C) Combustible High cost Low stiffness
Cement matrix	High compressive strength (to 100 MPa) Higher resistance to high temperatures than epoxy resins No plastic behaviour at high temperatures Incombustible Low cost High stiffness	Low tensile strength (to 10 MPa) Very low cooperation/no cooperation with FRP Low chemical resistance Low bending strength

**Table 5:** Influence of FRP composites on the behaviour of concrete.

Compressive Strength R_c_ as a Reference (MPa)	Laminate Type	Matrix	FRP Layers	Compressive Strength of Reinforced Specimen in Comparison to the Reference (%)	Reference	Type of Concrete
64.91	CFRP	ER	1	+33	[48]	High-performance concrete
40.32				+99	[49]	Fibre-reinforced normal concrete
81.04		CM		+4	[50]	High-performance self-compacting fibre-reinforced concrete
31.74		ER		+128	[51]	Normal concrete
33.7			2	+33	[52]	Reinforced normal concrete
			4	+71		
136			1	+29	[53]	Ultra-high performance fibre-reinforced concrete
			5	+55		
21.18			1	+80	[54]	Lightweight aggregate concrete
			3	+155		
38.83			1	+46		
			3	+120		
15.45			1	+236		
			3	+407		
64.4			1	+22	[55]	High-strength concrete
			2	+64		
			3	+99		
43.4			1	+20	[56]	Ready-mixed normal concrete
			3	+97		
	BFRP		1	+2		
			3	+3		
55.8			2	+0.8	[57]	Normal concrete
			4	+38		
			6	+69		
56.27			2	+41		High-performance concrete
76.98			4	+92		
94.57			6	+136		
26.26	GFRP		1	+8	[58]	Low-performance concrete
			2	+18		
136			5	+35	[53]	Ultra-high performance fibre-reinforced concrete
			9	+45		
27.2			2	+220	[59]	Low-performance concrete
			3	370		
44	AFRP		1	+242	[60]	Normal concrete
69.5			1	+49	[61]	Recycled tyre rubber concrete
			2	+109		
63.7			3	+116		
69.5			4	+180		
23.8			1	+99		
23.9			2	+196		
23.8			3	+296		
25.4			4	+335		
7.1			1	+251		
7.2			2	+450		
			3	+719		
7.8			4	+812		
110.3			4	+27	[62]	Ultra-high performance concrete
100.2			4	+64		
113.8			3	+16	[63]	Ultra-high performance concrete
113.8			4	+39		
113.8			6	+39		
23.1			1	+196	[64]	Low-performance concrete
85.7			6	+94	[65]	High-performance concrete

Note: ER—epoxy resin, CM—cement matrix.

**Table 6:** Technological, material, and environmental parameters and their major influence on the efficiency of reinforcing concrete structures with FRP laminates.

Parameter	Importance
Type of FRP	As fibre strength increases, the load-bearing capacity of a reinforced concrete structure increases.
Number of FRP layers	As the number of FRP laminates increases, the load-bearing capacity of a reinforced concrete structure increases.
Direction of fibres	Arranging fibres parallel to the tensile stresses increases the load capacity of the composite structure.
Different fibres used in the laminates	Combining different FRP fibres in multilayer laminates is possible and does not adversely affect the structure.
Matrix	The use of resins is recommended (especially epoxy resins). In the case of reinforcing low-performance concrete, cement mortar could be used, but the load-bearing efficiency, due to adhesion properties, in this case, is low.
Lamination process	The high quality of carried out work, including the correct reinforcement of the concrete substrate, accurate venting of the resin, and the correct adhesion of the laminate to the concrete surface guarantees good performance of the structure in accordance with the reinforcement design.
Type of concrete	As the strength of the concrete increases, the reinforcement efficiency decreases. It is recommended that concretes with low compressive strength should be reinforced with resin. Especially, concrete elements with low compressive strength may be reinforced with the use of composite meshes and a cement mortar (or matrix).
Type of concrete surface	The sandblasting, grinding, and shot blasting of concrete surfaces affect the load-bearing capacity of a reinforced element in comparison to an unprepared concrete surface at the level of several percentages. It has been noted in the literature that the connection between the FRP and the grinded concrete surface was the most favourable.
Preparation of concrete substrate	The concrete substrate must be clean, completely dry, and free from dirt and cement milk. It is widely recommended to prepare the concrete surface using mechanical treatment as it improves the adhesion of FRP to the concrete substrate. Epoxy resin has a greater possibility of penetrating into the concrete, thus, increasing the total contact area. The best results can be achieved by using a grinded concrete surface.
Geometry of elements for retrofitting	FRP laminate creates a coating that adjusts and adheres to the existing geometry of the element being reinforced. Due to this, most concrete elements, considering their shape, can be reinforced by this method. It is especially useful in strengthening objects that are several dozen or more years old. However, the trend in designing structures already using laminates is beginning to become noticeable.
Performance conditions	The lamination process should take place under positive temperature conditions and with low humidity.
Temperature	The temperature during the lamination process should be between +10 °C to +40 °C. As the ambient temperature rises, the use time of the resin and the inorganic matrix is shortened due to their accelerated fixation.
Insolation	High insolation, dark surfaces that do not reflect radiation and excessive heating of the structure may affect the achievement of the glass transition zone by the organic matrix, beyond which the matrix begins to deteriorate.
Humidity	The humidity of concrete substrate should be not more than 5% by weight. High humidity has a negative effect on the penetration depth of resin and cement mortar.
Design of new elements	By appropriate selection of FRP laminates, it is possible to make slender elements with smaller cross-sections. Advanced software allows to accurately determine the number of laminate layers. Numerous experimental studies are also helpful.
Fire protection	In the case of FRP laminates with epoxy resin, it is recommended to protect them against high temperatures by using insulation or cement matrix to confine the composite structure. FRP laminates with cement matrix do not need additional fire protection if the cement matrix has an appropriate width.

**Table 7:** Reduction factors and long-term stress limitation factors.

**Durability Reduction Factors**			
**Regulation**	**CFRP**	**GFRP**	**AFRP**
ACI 440.1R-15 [75]	0.95 *f_fu_*	0.75 *f_fu_*	0.85 *f_fu_*
**Long-Term Stress Limitation Factors**			
ACI 440.1R-15 [75]	0.55 *f_fu_*	0.20 *f_fu_*	0.30 *f_fu_*
